# Protein Expression of NEK2, JMJD4, and REST in Clear Cell Renal Cell Carcinoma (ccRCC): Clinical, Pathological, and Prognostic Findings

**DOI:** 10.30699/IJP.2023.1974154.3022

**Published:** 2023-06-20

**Authors:** Walid S H Elsayed, Ola A Harb, Mohamed Ali Alabiad, Rema H Faraj Saad, Amal Anbaig, Mohammed Alorini, Rehab Hemeda, Mohamed Negm, Loay M Gertallah, Waleed A Abdelhady, Ramadan M Ali

**Affiliations:** 1 *Department of Pathology, Faculty of Medicine, Zagazig University, Zagazig, Egypt*; 2 *Department of Pathology, Faculty of Medicine, University of Benghazi, Benghazi, Libya*; 3 *Department of Basic Medical Sciences, Unaizah College of Medicine and Medical Sciences, Qassim University, Unaizah, Kingdom of Saudi Arabia *; 4 *Department of Clinical Oncology and Nuclear Medicine, Faculty of Medicine, Zagazig University, Zagazig, Egypt*; 5 *Department of General Surgery, Faculty of Medicine, Zagazig University Zagazig, Egypt*

**Keywords:** JMJD4, NEK2, Prognosis, Renal cell carcinoma, REST

## Abstract

**Background & Objective:**

Cells of renal cell carcinoma (RCC) are resistant to the most currently used chemotherapeutic agents and targeted therapies; hence, we evaluated the expression of NEK2, JMJD4, and REST in cases of clear cell renal cell carcinoma (ccRCC) and benign adjacent tissues of kidney to detect associations between their expression and clinicopathological features, prognostic data, tumor recurrence, and survival rates.

**Methods:**

We collected 200 samples including tumoral and adjacent non-neoplastic tissues related to 100 ccRCC patients. All samples were evaluated for the expression of NEK2, JMJD4, and REST, and the patients were followed up for about 5 years. Tumor recurrence and survival data were documented and analyzed.

**Results:**

NEK2 and JMJD4 expression showed increase in ccRCC tissues (*P*=0.002 and 0.006), while REST was downregulated (*P*<0.001). The elevated expression of NEK2 was positively related ro the tumor size (*P*=0.015), higher grades (*P*=0.002), higher stages (*P*=0.013), distant spread (*P*=0.004), tumor recurrence, shorter progression-free survival (PFS) rate, and overall survival (OS) rate (*P*<0.001). Likewise, the high expression of JMJD4 showed positive correlation with the tumor size (*P*=0.047), higher grades (*P*=0.003), higher stages (*P*=0.043), distant spread (*P*=0.001), tumor recurrence, shorter PFS rate, and OS rate (*P*<0.001). Conversely, low expression of REST demonstrated positive relationship with the tumor size, higher grades, higher stages, distant spread, tumor recurrence, and shorter PFS and OS rates (*P*<0.001).

**Conclusion:**

Overexpression of NEK2 and JMJD4 and downregulation of REST may be noted in malignant renal tissues compared to benign renal tissues and may be correlated with unfavorable pathological findings, poor clinical parameters, and poor patient outcomes.

## Introduction

Renal cell carcinoma (RCC) is a common aggressive urological malignancy with an increasing incidence each year (1). Clear cell renal cell carcinoma (ccRCC) is the commonest subtype (2). The cells of RCC are resistant to the most currently used chemotherapeutic agents and targeted therapies; so there is an urgent need to detect novel effective diagnostic and prognostic biomarkers and targeted therapies to improve their prognosis (3).

NIMA (Never in Mitosis Gene A)-related kinase 2 (NEK2) is a protein that has a role in cell cycle (4)**.** The oncogenic functions of NEK2 have been recently discovered while many studies have reported that its expression increases in plethora of malignant tumors and is related to poor survival rates and unfavorable results (5). However, the association between the NEK2 expression and patients' prognosis in ccRCC cells has not been fully studied (6).

Histone modifications are essential epigenetic mechanisms that could modulate gene expression (7). Histone demethylases are common modifying enzymes that have roles in histone modifications (8). Jumonji-C domain-containing histone-demethylase 4 (JMJD4) is a newly discovered histone demethylase that has a role in carcinogenesis and its expression has been studied in many cancers, though it has not been sufficiently clarified in ccRCC. 

Repressor element-1 silencing transcription factor (REST) is a transcription factor that was known to suppress transcription of genes (9, 10).

It was recently pointed out that REST inhibits tumor occurrence and thus was hypothesized to have an oncosuppressive role in ccRCC (11).

The aim was to evaluate the expression of NEK2, JMJD4, and REST in tissues of ccRCC and benign kidney to detect the relationship between their expression and clinical, pathological, prognostic, recurrence, and survival findings.

## Methods and Materials

**Patients and Samples**

We included 100 patients with ccRCC who had underwent nephrectomy in Zagazig University Hospitals, Department of General Surgery, from March 2016 to March 2020.

Processing, diagnosis, subtyping, classification, grading, and staging of resected tissues were performed in Pathology Department.

**Inclusion and Exclusion Criteria**

Patients with a confirmed diagnosis of ccRCC at different grades and stages who had complete pathological and clinical data, were included in the study. Whereas, patients who had underwent preoperative radiation therapy and chemotherapy, patients with autoimmune diseases, and kidney infection or failure, patients with a malignant tumor of other organs, and patients with incomplete data were excluded. 

After considering inclusion criteria, 200 samples were recovered from tumor tissues (100 samples), and adjacent non-neoplastic tissues (100 samples) of patients with ccRCC. We collected all clinical and pathological findings from them and followed them up in the Clinical Department of Oncology and Nuclear Medicine and the Medical Oncology Department for approximately 5 years, where recurrence and survival data were documented and analyzed.

The local institutional review board of the Faculty of Medicine of Zagazig University approved this study. We obtained written informed consent from all included patients.

**Immunohistochemistry**

 Immunohistochemistry (IHC) was performed on 200 samples, including 100 from ccRCC tissues and 100 benign kidney tissues of the same patients as previously described (12-15). Antigens were taken out of the tissue sections by heating (16, 17). The sections were then treated with anti-NEK2 primary antibody (ab227958, 1:200; Abcam, Cambridge, UK), anti-JMJD4 primary antibody (AP1030a, Abcepta, San Diego, CA, USA), and anti-REST primary antibody (ab21635, Abcam, Cambridge, MA). The sections were then rinsed with phosphate-buffered saline and treated for two hours with a secondary antibody that matched the primary antibody (13, 14). The avidin-biotin compound was applied to the sections for 20 minutes (18-20). The slices were then washed again, and the immunoreactivity was determined by DAB (3, 3'-diaminobenzidine) staining and hematoxylin counterstain. Immunoreactivity was demonstrated by a brown stain (21-24).

**Evaluation of NEK2, JMJD4, and REST Expression in Stained Tissues of ccRCC and Benign Kidney Tissues**

We evaluated the expression of NEK2, JMJD4, and REST by evaluating the intensity scores (0-negative, 1-weak, 2-moderate, 3-strong) and multiplying them by extent scores (0∼5%-1, 5∼25%-2, 25∼75%-3, >75%-4), which resulted in a final staining score of 0-12. We considered scores 0–7 as low expression and scores 8–12 as high expression.

**Statistical Analysis**

We collected and analyzed data using the SPSS software version 21.0 (Chicago, IL, USA) and Prism 8.0 (GraphPad Software, San Diego, CA, USA). We presented the data with normal distribution as means ± standard deviation (SD).

We analyzed categorical data using the Chi-square test or Fisher's exact test. We also used Pearson correlation coefficient to analyze the association between the expression of markers and clinicopathological data. Furthermore, we performed univariate and multivariate analyses. We also analyzed progression-free survival (PFS) and overall survival (OS) rates using logrank test and Kaplan–Meier survival curve. We considered *P*<0.05 as a statistically significant value. 

## Results

The clinical findings of included patients were analyzed; based on the records, 82% of patients were male, with 62% of them being over 55 years old. Moreover, 24% of the patients were grade I, 36% were grade II, 30% were grade III, and 10% were grade IV (Table 1).

**NEK2 Expression in Stained Tissues and Association with Clinicopathological Findings**

**Fig. 1:**
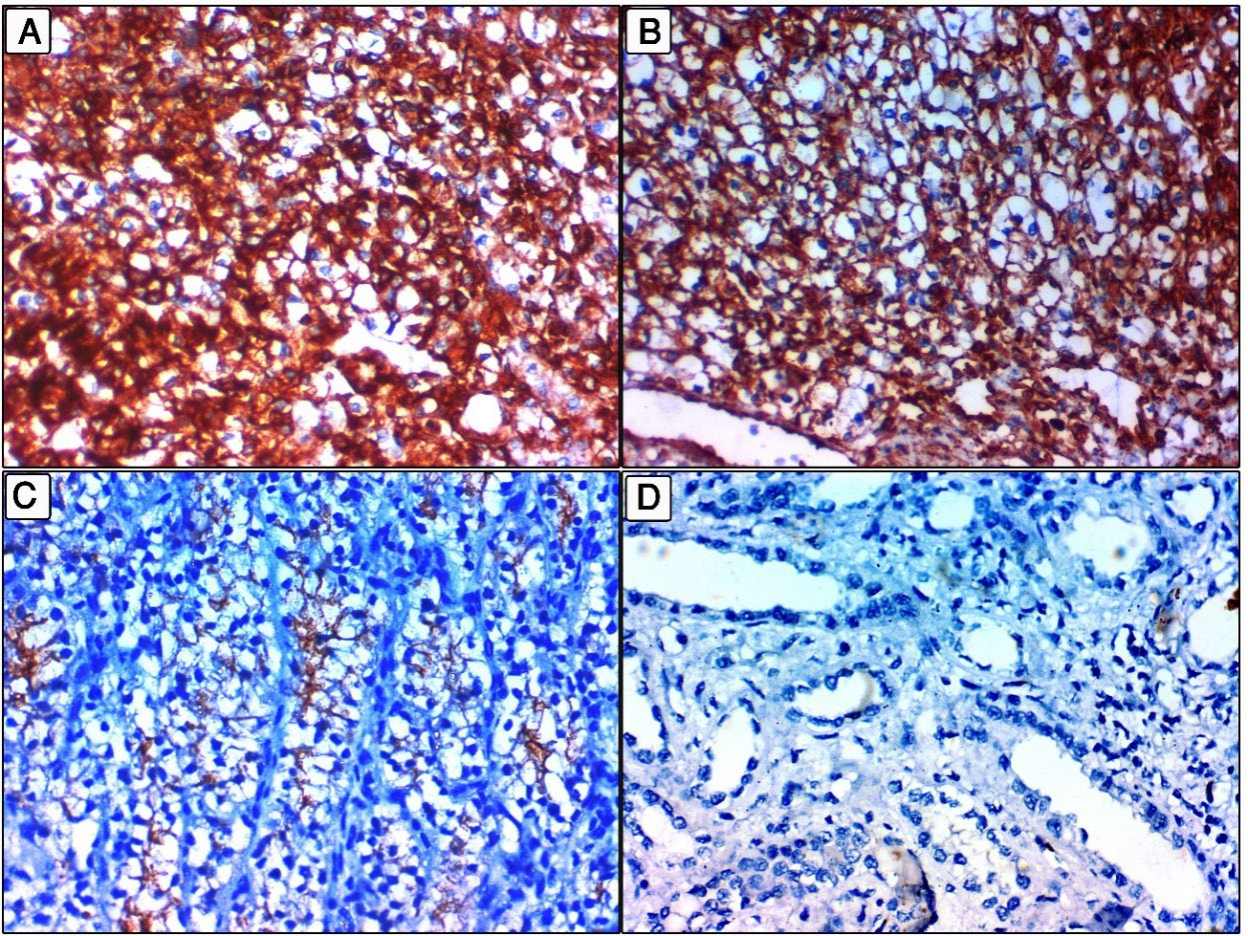
NEK2 expression in clear cell renal cell carcinoma (ccRCC). A: Cytoplasmic overexpression in a high grade ccRCC, stage IV (400x). B: Cytoplasmic overexpression in high grade ccRCC, stage III, (400x). C: Decreased cytoplasmic expression in a low grade ccRCC, stage I (400x). D: Negative cytoplasmic expression in non-neoplastic renal tissues

High expression of NEK2 was observed in 47.1% of all 200 included samples. NEK2 expression was increased in 58% of ccRCC tissues and in 20% of benign kidney tissues (*P*=0.002). NEK2 elevated levels were positively associated with big tumor mass (*P*=0.015), higher grades (*P*=0.002), advanced stages (*P*=0.013), and distant spread (*P*=0.004). No significant association was found between its expression and patient age or gender (Tables 2, 3, and 4, Figure 1).

**Table 1:** Demographic, clinical data and pathological features of the patients with clear cell renal cell carcinoma (ccRCC) patients

		Total samples N=200	cc-RCC N=100
Age group	<55y	38(38.0%)	38(38.0%)
	>55y	62 (62.0%)	62 (62.0%)
Histopathology of samples	ccRCC	100 (50%)	
	Normal	100 (50%)	
Sex	Male	82 (82%)	82 (82%)
	Female	18 (18%)	18 (18%)
Grade	1	24 (24.0%)	24 (24.0%)
	2	36 (36.0%)	36 (36.0%)
	3	30 (30.0%)	30 (30.0%)
	4	10 (10.0%)	10 (10.0%)
Size	<7cm	26 (26.0%)	26 (26.0%)
	>7cm	74 (74.0%)	74 (74.0%)
T	1	26 (26.0%)	26 (26.0%)
	2	32 (32.0%)	32 (32.0%)
	3	26 (26.0%)	26 (26.0%)
	4	16 (16.0%)	16 (16.0%)
N	0	56 (56.0%)	56 (56.0%)
	1	44(44.0%)	44(44.0%)
M	0	78(78.0%)	78(78.0%)
	1	22 (22.0%)	22 (22.0%)
Stage	I	22 (22.0%)	22 (22.0%)
	II	36 (36.0%)	36 (36.0%)
	III	22 (22.0%)	22 (22.0%)
	IV	20 (20.0%)	20 (20.0%)

**JMJD4 Expression in Stained Tissues and Association with Clinicopathological Findings**

**Fig. 2:**
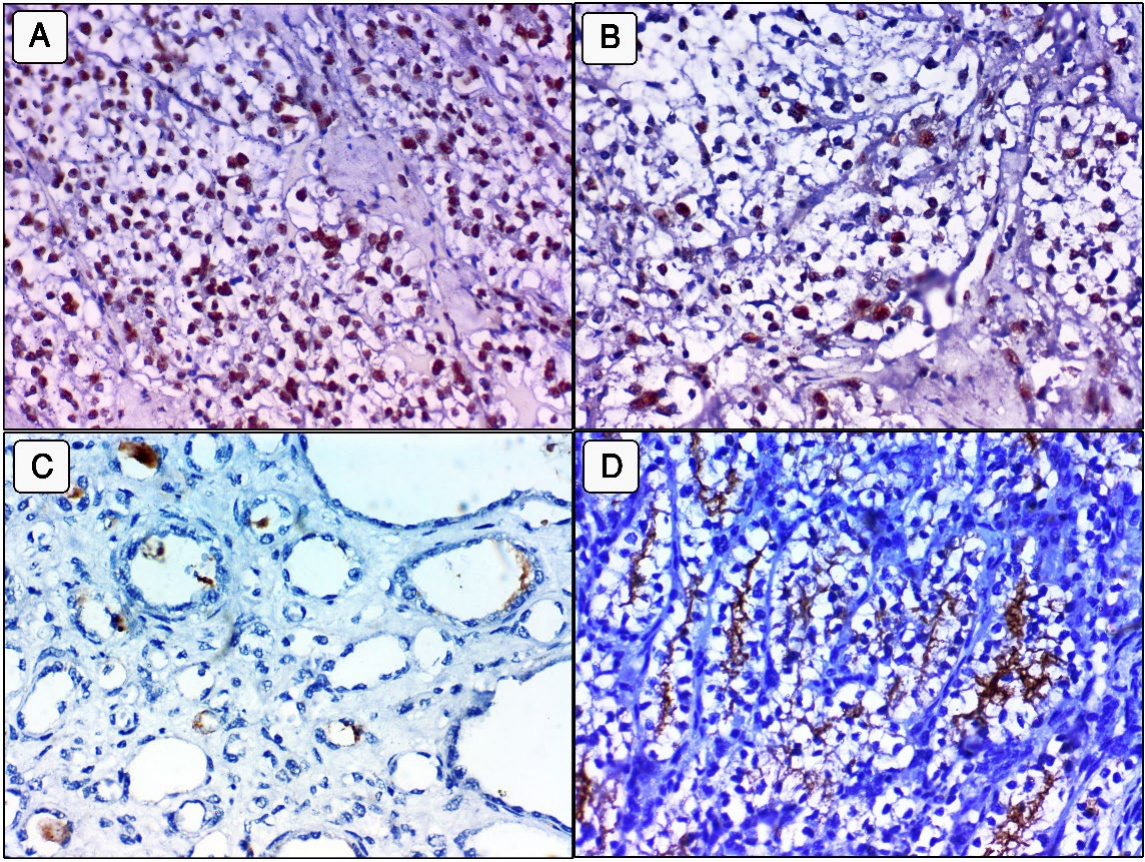
JMJD4 expression in clear cell renal cell carcinoma (ccRCC). A: Nuclear overexpression in a high grade ccRCC, stage IV (400x). B: Nuclear overexpression in a high grade ccRCC, stage III, (400x). C: Decreased nuclear expression in a low grade ccRCC, stage I, (400x). D: No nuclear expression in a non-neoplastic renal tissues

Likewise, high expression of JMJD4 was seen in 47.1% of the 200 included samples. JMJD4 expression was upregulated in 62% of ccRCC tissues and in 10% of benign kidney tissues (*P*=0.006). Elevated levels of JMJD4 was positively correlated with big tumor mass (*P*=0.047), higher grades (*P*=0.003), higher stages (*P*=0.043), and distant spread (*P*=0.001). No significant association was found between its expression and patient age or gender (Tables 2, 3, and 4, Figure 2). 

**REST Expression in Stained Tissues and Association with Clinicopathological Findings**

**Fig. 3:**
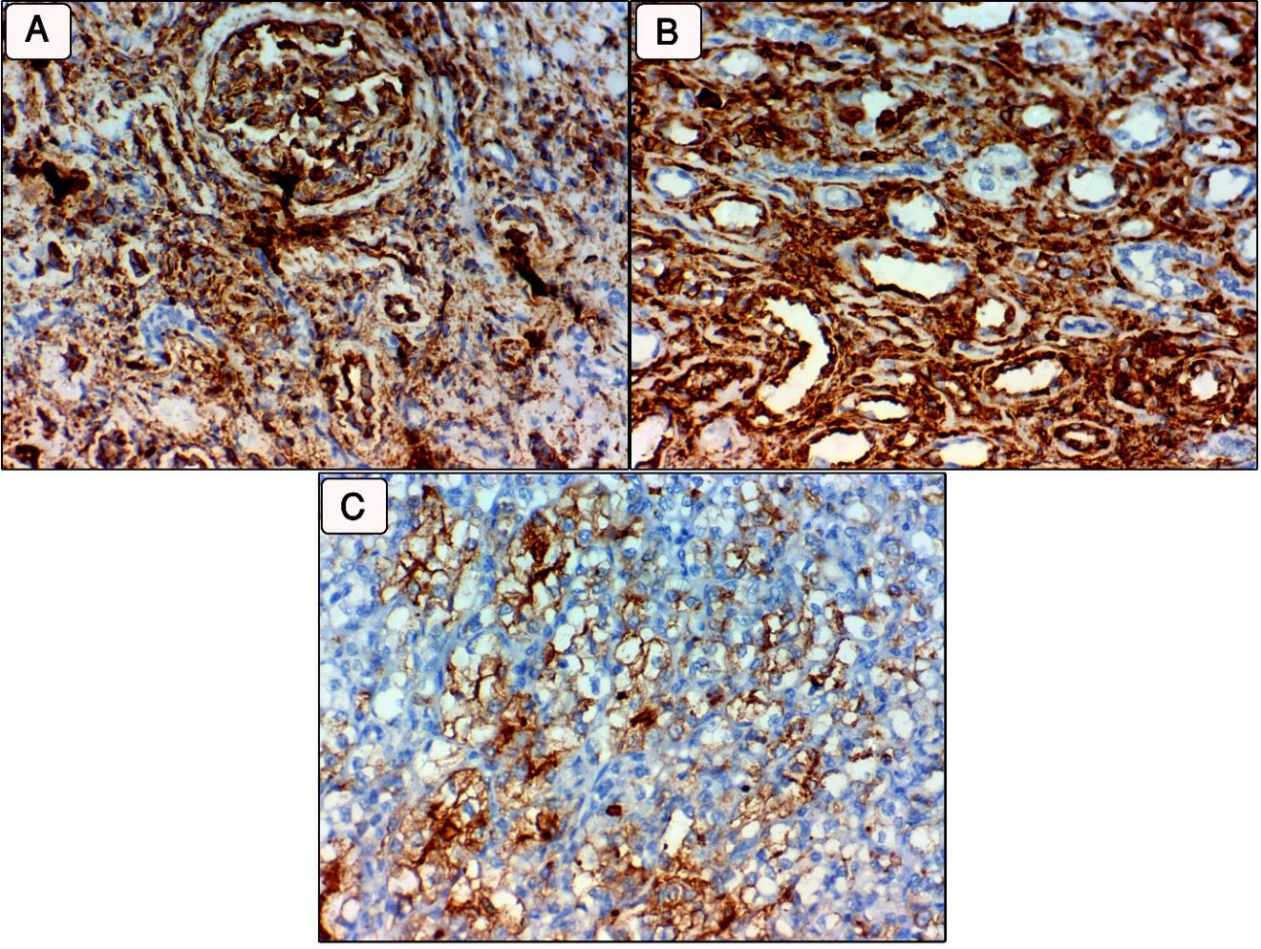
REST expression in clear cell renal cell carcinoma (ccRCC). (A& B: High expression in benign non-neoplastic renal tissues. C: Low expression in high grade ccRCC, stage IV (400x)

On the contrary, low expression of REST was observed in 76% of cc-RCC tissues and 26% of benign kidney tissues. Low expression of REST was related to big tumor mass, higher grades, higher stages, and the p-value of the four factors is the same and equals P<0.001. No significant association was found between its expression and patient age or gender (Tables 2, 3, and 4, Figure 3).

Based on the findings, there was a direct correlation between NEK2 and JMJD4 expression (r=+0.422, *P*=0.005), an indirect correlation between NEK2 and REST expression (r=-0.316, *P*=0.002), and an indirect correlation between JMJD4 and REST expression (r=-0.396, *P*=0.042).

**Survival Analysis**

**Fig. 4.**
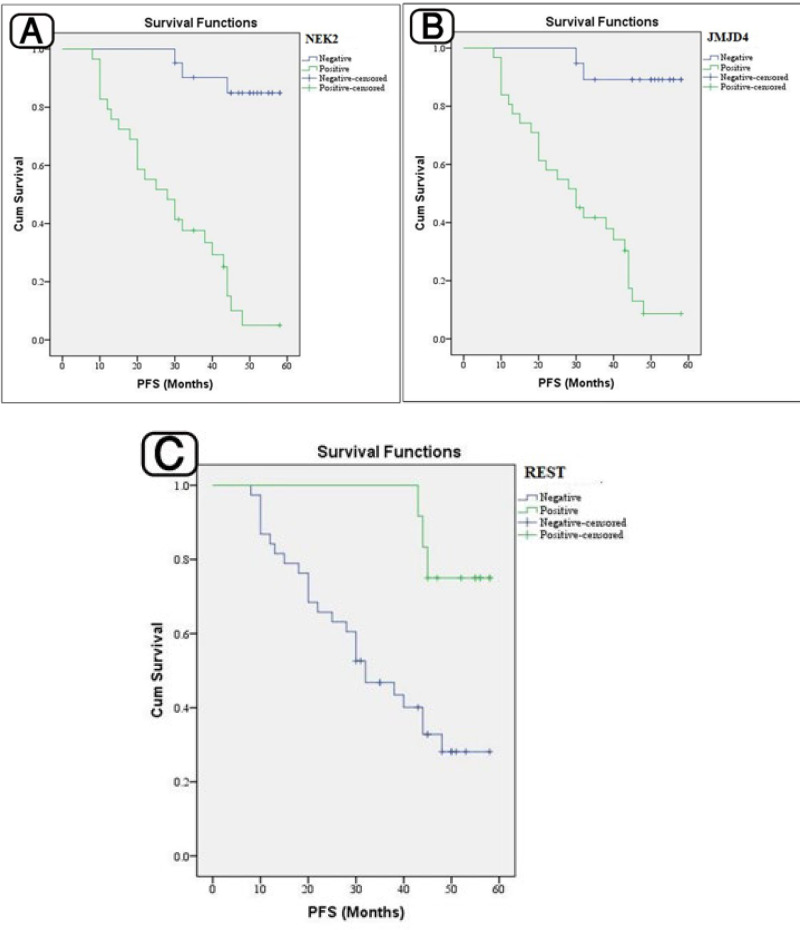
**A:** Progression free survival (PFS) rates assessed according to the NEK2 expression.** B: **PFS rate assessed according to the JMJD4 expression. C: PFS rate assessed according to the REST expression

**Fig. 5:**
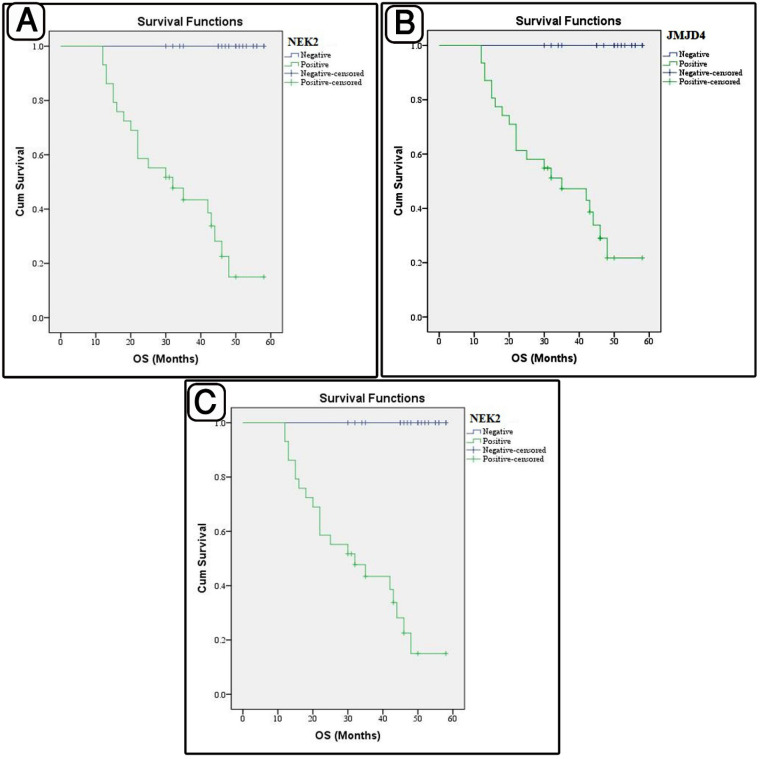
**A: **Overall survival (OS) rate assessed according to the NEK2 expression. B: OS rate assessed according to the JMJD4 expression. C: OS rate assessed according to the REST expression

After a median follow-up period of 45.5 months (12-58), 42% of the patients died. The 5-year overall survival rate was 57% (95% CI) (Tables 5 and 6, Figures 4 and 5). The 5-year PFS rate was 40% (95% CI), while the median PFS was 45 months (95% CI).

**Expression of NEK2, JMJD4, and REST in Stained Tissues and Their Association with Prognostic and Follow-up Findings**

High levels of NEK2 and JMJD4 were both related to tumor recurrence, and shorter RFS and OS rates (*P*<0.001). While the association of low levels of REST was found with these factors, that are, tumor recurrence, and shorter RFS and OS rates (*P***<**0.001).

**Table 2:** NEK2, JMJD4 and REST expression in stained tissues

		Total N=100 N (%)	CCRCC N=100	NORMAL N=100
			N (%)	N (%)
**NEK2**	Low	52.9(52.9%)	42 (42.0%)	80 (80.0%)
	High	47.1 (47.1%)	58 (58.0%)	20 (20.0%)
JMJD4	Low	52.9 (52.9%)	38 (38.0%)	90 (90.0%)
	High	47.1 (47.1%)	62 (62.0%)	10 (10.0%)
REST	Low	61.4 (61.4%)	76 (76.0%)	25 (25.0%)
	High	38.6 (38.6%)	24 (24.0%)	75 (75.0%)

**Table 3:** NEK2, JMJD4 and REST expression in stained tissues and association with histopathology, age and sex of the patients

		**NEK2**			*P*	JMJD4		*P*	REST		*P*
		Low N=52.9		High N=47.1		Low N=52.9	High N=47.1		Low N=61.4	High N=38.6	
Age group	<55y		27 (27.0%)	42 (42%)	0.175	32.4 (32.4%)	36.4 (36.4%)	0.729	14 (32.6%)	10 (37.0%)	0.701
	>55y		73 (73.0%)	58 (58%)		67.6 (67.6%)	63.6 (63.6%)		29 (67.4%)	17 (63.0%)	
Histopathology	cc-RCC		58 (58%)	42 (42%)	0.002	38 (38%)	62 (62%)	0.006	76 (76%)	24 (24%)	<0.001
	Normal		80 (80%)	20 (20%)		90 (90%)	10 (6.1%)		25 (25%)	75 (75%)	
Sex	Male		73 (73.0%)	90 (90.9%)	0.054	73 (73.0%)	90.9 (90.9%)	0.054	36 (83.7%)	77.8 (77.8%)	0.534
	Female		27 (27.0%)	9.1 (9.1%)		27 (27.0%)	9.1 (9.1%)		16.3 (16.3%)	22.2 (22.2%)	

**Table 4:** NEK2, JMJD4 and REST expression in stained tissues and association with clinicopathological data

RCC		**NEK2**		*P*	JMJD4		*P*	REST		P
		Low	High		Low	High		Low	High	
		N=58	N=42		N=38	N=62		N=76	N=24	
Age group	<55y	(47.4%)	(32.3%)	0.285	(42.9%)	(34.5%)	0.547	(36.8%)	(41.7%)	0.51
	>55y	(52.6%)	(67.7%)		(57.1%)	(65.5%)		(63.2%)	(58.3%)	
Sex	Male	(73.7%)	(90.3%)	0.119	(76.2%)	(89.7%)	0.255	(81.6%)	(91.7%)	0.406
	Female	(26.3%)	(9.7%)		(23.8%)	(10.3%)		7 (18.4%)	1 (8.3%)	
Grade	1	(47.4%)	(9.7%)	0.002	(42.9%)	(10.3%)	0.003	(5.3%)	(83.3%)	<0.001
	2	(36.8%)	(35.5%)		(42.9%)	(31.0%)		(42.1%)	(16.7%)	
	3	(15.8%)	(38.7%)		(14.3%)	(41.4%)		(39.5%)	(0.0%)	
	4	0 (0.0%)	(16.1%)		0 (0.0%)	(17.2%)		(13.2%)	(0.0%)	
Size	<7cm	(36.8%)	(19.4%)	0.015	(33.3%)	(20.7%)	0.047	(7.9%)	(83.3%)	<0.001
	>7cm	(63.2%)	(80.6%)		(66.7%)	(79.3%)		35 (92.1%)	2 (16.7%)	
T	1	(36.8%)	(19.4%)	0.046	(33.3%)	(20.7%)	0.018	(7.9%)	(83.3%)	<0.001
	2	(42.1%)	(25.8%)		(47.6%)	(20.7%)		(36.8%)	(16.7%)	
	3	(21.1%)	(29.0%)		(19.0%)	(31.0%)		(34.2%)	(0.0%)	
	4	(0.0%)	(25.8%)		0 (0.0%)	(27.6%)		(21.1%)	0 (0.0%)	
N	0	(78.9%)	(41.9%)	0.011	(81.0%)	(37.9%)	0.022	(42.1%)	(100.0%)	<0.001
	1	(21.1%)	(58.1%)		(19.0%)	(62.1%)		22 (57.9%)	0 (0.0%)	
M	0	(100.0%)	(64.5%)	0.004	(100.0%)	(62.1%)	0.001	(71.1%)	(100.0%)	0.035
	1	0 (0.0%)	(35.5%)		0 (0.0%)	(37.9%)		(28.9%)	0 (0.0%)	
Stage	I	(36.8%)	(12.9%)	0.013	(33.3%)	(13.8%)	0.043	(2.6%)	(83.3%)	<0.001
	II	(42.1%)	(32.3%)		(47.6%)	(27.6%)		(42.1%)	(16.7%)	
	III	(21.1%)	(22.6%)		(19.0%)	(24.1%)		(28.9%)	0 (0.0%)	
	IV	0 (0.0%)	(32.3%)		0 (0.0%)	(34.5%)		(26.3%)	0 (0.0%)	

**Table 5:** NEK2, JMJD4 and REST expression in stained tissues and association with progression and survival rates of the ccRCC patients

ccRCC		Total N=100	NEK2		*P*	JMJD4		*P*	REST		*P*
			Low N=58	High N=42		Low N=38	High N=62		Low N=76	High N=24	
Progression	Absent	44 (44.0%)	18 (85.7%)	13.8 (13.8%)	**<0.001**	89.5 (89.5%)	5 (16.1%)	**<0.001**	34.2 (34.2%)	75 (75.0%)	**0.013**
	Present	56 (56.0%)	3 (14.3%)	86.2 (86.2%)		10.5 (10.5%)	83.9 (83.9%)		65.8 (65.8%)	25 (25.0%)	
Survival status	Censored	58 (58.0%)	21 (100.0%)	27.6 (27.6%)	**<0.001**	100 (100.0%)	32.3 (32.3%)	**<0.001**	50 (50.0%)	83.3 (83.3%)	**0.041**
	Died	42 (42.0%)	0 (0.0%)	72.4 (72.4%)		0 (0.0%)	67.7 (67.7%)		50 (50.0%)	16. 7 (16.7%)	

**Table 6:** Univariate analysis of overall and Progression -Free Survival in relation to some studied parameters. NEK2, JMJD4 and REST

Variables		5-year Overall survival Rate (%)	*P*	5-year Progression-Free survival Rate (%)	*P*
Age group	<55y	56.4%	**0.748**	57.0%	**0.174**
	>55y	48.1%		29.3%	
Sex	Male	51.2%	**0.744**	36.3%	**0.368**
	Female	60%		60.0%	
Size	<7 Cm	59.3%	**0.249**	53.8%	**0.093**
	>7 Cm	55.4%		36.3%	
Grade	1	80%	**< 0.001**	83.3%	**< 0.001**
	2	63.5%		49.9%	
	3	29.6%		46.7%	
	4	20%		0.0%	
T	1	59.3%	**0.004**	53.8%	**< 0.001**
	2	74.5%		68.2%	
	3	53.8%		19.2%	
	4	25%		0.0%	
N	0	68.4%	**0.002**	63.2%	**< 0.001**
	1	39.8%		0.0%	
M	0	61.8%	**0.003**	49.9%	**< 0.001**
	1	24.2%		9.1%	
Stage	Stage I	71.1%	**0.005**	63.6%	**< 0.001**
	Stage II	65.5%		59.8%	
	Stage III	45.5%		24.2%	
	Stage IV	37.5%		0.0%	
NEK2	Negative	100%	**< 0.001**	84.9%	**< 0.001**
	Positive	15%		5%	
JMJD4	Negative	100%	**< 0.001**	89.4%	**< 0.001**
	Positive	21.7%		8.7%	
REST	Negative	42.2%	**0.017**	28.1%	**0.004**
	Positive	82.5%		75.0%	

## Discussion

In the current study, we evaluated NEK2 protein expression in ccRCC tissues and non-neoplastic renal tissues and found that it was increased in malignant tissues compared to the non-neoplastic renal tissues. Additionally, its expression was positively associated with larger tumor size, high grade, presence of lymph node metastases, and high stage. Furthermore, it was positively correlated with a high incidence of tumor recurrence and shorter RFS and OS rates.

NEK2 plays many roles in cancer progression as explained by Zhou *et al.* (25) in diffuse large B-cell lymphoma. NEK2 controls glycolysis in tumor cells. Its overexpression was associated with tumor progression and poor prognosis**.** Furthermore, reduced NEK2 expression was found to be associated with the inhibition of non-small cell lung cancer oncogenesis and spread (26). Additionally, NEK2 silencing was found to inactivate protein kinase B (PKB/Akt), decrease aerobic glycolysis, and stimulate autophagy that could inhibit malignant gastric cell growth (27)**.**

Our results showed that NEK2 expression was elevated in ccRCC tissues and its high expression was related to big tumor mass, higher grades, higher stages, distant spread, and shorter PFS and OS rates.

A few studies have evaluated the expression of NEK2 in ccRCC. Similar to our study, Wang *et al.* (6) showed high NEK2 expression in tissues of ccRCC, and that its elevated levels was related to unfavorable clinical outcomes and poor prognosis of patients with ccRCC.

NEK2 is a new prognostic biomarker and a therapeutic target in ccRCC, however since few studies have assessed its expression in RCC, in this study we evaluated the expression of other novel markers in ccRCC; JMJD4 and REST. 

JMJD4 usually plays a role in embryogenesis (28). Some studies have evaluated JMJD4 expression in cancer. For example, Ho *et al.* (29) demonstrated its prognostic role in colon cancer. In our study, high JMJD4 expression was observed in ccRCC tissues more than that in benign renal tissues, and its expression was associated with unfavorable clinical and prognostic findings. Yan *et al.* (3) displayed similar results on the roles of JMJD4 in ccRCC and its association with unfavorable outcomes. JMJD6 was found to be an oncogenic factor in many cancers, and MJD4 is considered one of its metabolites (28, 30-32). 

Yan *et al.* (3) showed that JMJD4 is a cancer promoter agent in ccRCC, promoting cancer cell invasion and progression. In the present study, we showed that NEK2 expression was positively associated with JMJD4 expression. The overexpression of both genes was more present in ccRCC tissues compared to benign renal tissues.

We evaluated the expression of another biomarker (i.e., REST) in ccRCC and non-neoplastic renal tissues and showed that REST was downregulated in ccRCC and its high expression was found in adjacent non-neoplastic renal tissues. Furthermore, REST expression was negatively correlated with high grade, high stag, and unfavorable survival rates.

Our results were in line with the results of Lv *et al.* (11), who demonstrated low REST expression in ccRCC; its expression decreased in larger tumor cells. Additionally, they showed that its low expression was negatively correlated with the patients’ survival.

Previous studies have also shown overexpression of REST in many types of tumors such as neuroblastoma, glioma, and medulloblastoma (33-35). 

In our study, REST expression showd a decrease in malignant tumors compared to the benign tissues. This result is inconsistent with those obtained from other similar studies performed on various cancer types. . The decrease in REST expression can be attributed to the fact that the role of REST in carcinogenesis is complex and depends on the cancer type. Recently, REST expression was found to be associated with cancer progression, though its exact role in carcinogenesis is uncertain (36).

One study demonstrated that decreased REST expression may initiate malignant transformation in the epithelial cells (37). REST regulates the growth and survival of ovarian cancer cells by regulating mTOR signaling pathways (38). In addition, REST expression is positively associated with increased malignant invasion, stages of cancer, and spread to lymph nodes. Patients with medulloblastoma with high expression of REST have worse overall survival (39). However, we showed that REST overexpression in ccRCC patients is associated with favorable patient survival rates.

## Conclusion

To sum up, we evaluated the expression of NEK2, JMJD4, and REST tissue proteins in ccRCC and benign kidney tissues. We showed that REST expression was inversely associated with NEK2 and JMJD4 expression in ccRCC and benign renal tissues. Furthermore, in our study, overexpression of NEK2 and JMJD4 and downregulation of REST were observed in malignant than benign renal tissues and were related to unfavorable pathological findings, poor clinical parameters, and poor patient outcomes.

Our findings provided insight into prognosis of the patients with ccRCC and discovery of new and potential therapeutic targets. Further studies are needed to explain the associated mechanisms of action of these biomarkers in the process of cancer progression, in order to provide better treatment strategies for patients with ccRCC.

## Limitations

small sample size, shorter follow-up period, and evaluation of markers’ expression using only immunohistochemistry without molecular assessment were limitations of the present study.

## Conflict of Interest

None.
